# Regulation of osteoclasts by membrane-derived lipid mediators

**DOI:** 10.1007/s00018-012-1238-4

**Published:** 2013-01-08

**Authors:** Tsukasa Oikawa, Yukiko Kuroda, Koichi Matsuo

**Affiliations:** Laboratory of Cell and Tissue Biology, School of Medicine, Keio University, 35 Shinanomachi, Shinjuku-ku, Tokyo, 160-8582 Japan

**Keywords:** IP3, PI3-kinase, Circumferential podosome, Fusion-competent protrusion, Actin ring, Ruffled border

## Abstract

Osteoclasts are bone-resorbing cells of monocytic origin. An imbalance between bone formation and resorption can lead to osteoporosis or osteopetrosis. Osteoclastogenesis is triggered by RANKL- and IP3-induced Ca^2+^ influx followed by activation of NFATc1, a master transcription factor for osteoclastogenic gene regulation. During differentiation, osteoclasts undergo cytoskeletal remodeling to migrate and attach to the bone surface. Simultaneously, they fuse with each other to form multinucleated cells. These processes require PI3-kinase-dependent cytoskeletal protein activation to initiate cytoskeletal remodeling, resulting in the formation of circumferential podosomes and fusion-competent protrusions. In multinucleated osteoclasts, circumferential podosomes mature into stabilized actin rings, which enables the formation of a ruffled border where intensive membrane trafficking is executed. Membrane lipids, especially phosphoinositides, are key signaling molecules that regulate osteoclast morphology and act as second messengers and docking sites for multiple important effectors. We examine the critical roles of phosphoinositides in the signaling cascades that regulate osteoclast functions.

## Introduction

Osteoclasts are a unique cell type highly specialized for resorbing bone matrix. Hyperactivation of osteoclasts can result in bone-degenerative disorders such as osteoporosis and osteolytic bone metastasis, while lack or hypoactivation causes osteopetrosis. Active osteoclasts are polykaryons formed by cell–cell fusion of highly motile progenitors of the monocyte–macrophage lineage. For bone resorption, osteoclasts attach firmly to the bone surface by forming stable actin rings. Through the area enclosed by actin rings, osteoclasts secrete digestive acids and proteases and transport degraded matrix components by endocytosis/transcytosis into the cell and to the apical surface. In this way, osteoclasts facilitate bone remodeling and the recycling of bone nutrients, particularly calcium and phosphates.

Osteoclast precursors on the bone surface are stimulated by macrophage colony-stimulating factor (M-CSF) and receptor activator of NF-κB ligand (RANKL) produced by osteoblast lineage cells, resulting in the activation of the immediate early transcription factors NF-κB and c-Fos. These transcription factors are essential for the activation of signaling cascades that drive osteoclastogenesis [1]. Activation of RANK is an early event in osteoclastogenesis, leading to phospholipase C (PLC) activation, membrane hydrolysis of phosphatidylinositol 4,5-bisphosphate (PIP2) to form diacylglycerol and inositol-1,4,5-trisphosphate (IP3), IP3-mediated Ca^2+^ release and the activation of the Ca^2+^-dependent phosphatase calcineurin. Activated calcineurin dephosphorylates and thereby activates nuclear factor of activated T cells cytoplasmic 1 (NFATc1) [2–4], a transcription factor that activates the expression of multiple osteoclastogenic genes, including the membrane fusion promoter (fusogen) DC-STAMP, the actin ring component β3 integrin and the bone degrading hydrolases tartrate-resistant acid phosphatase (TRAP) and cathepsin K [5].

In addition, osteoclast differentiation depends on dramatic changes in cytoskeletal dynamics. Activated osteoclast precursors develop columnar actin puncta, known as podosomes, at the ventral surface [6, 7]. In the early phase of osteoclast differentiation, these actin puncta organize into dynamic rings, and as the cells fuse, these circumferential podosomes eventually mature into stabilized structures known as actin rings that adhere to the bone and isolate the contact site between the osteoclast ruffled membrane and the bone surface from the extracellular fluid [6]. Efficient bone resorption is then achieved through the secretion of protons and hydrolases, including TRAP and protease cathepsin K, at the ruffled border formed inside these belts [8], and concomitant incorporation of the degraded materials by endocytosis.

Cell–cell fusion and vesicle-ruffled border fusion both require intricate orchestration of the plasma membrane and vesicular membranes, involving signaling cascades mediated by membrane lipids. Membrane lipids, including phosphoinositides (PIs), contribute to a wide range of basic biological processes, such as polarity formation, chemotaxis, intercellular trafficking, and cytokinesis [9]. PIs are essential not only as membrane constituents in Eukaryotes and as precursors of second messengers like IP3 but also serve as specialized membrane docking sites for effectors of various signaling cascades [9]. Accumulating evidence suggests that PIs and PI-interacting proteins such as Rho, Arf, and Rab small GTPases function as modulators of osteoclast differentiation [10, 11]. In this review, we focus on recent advances in understanding the regulation of osteoclastogenesis by membrane-derived lipid mediators.

## Upregulation of intracellular Ca^2+^ concentration by IP3 activates NFATc1, a master transcription factor for osteoclastogenesis

Sustained activation of transcription factor NFATc1 is a crucial step in osteoclast differentiation and maturation. Forced expression of NFATc1 in bone marrow macrophages induces osteoclast differentiation, while NFATc1-deficient embryonic stem cells fail to differentiate into osteoclasts following stimulation with RANKL [3, 12]. The canonical mechanism of NFATc1 activation is through dephosphorylation by calcineurin, a Ca^2+^/calmodulin-dependent phosphatase, and subsequent nuclear translocation [13]. During osteoclastogenesis, intracellular Ca^2+^ levels oscillate in response to RANKL stimulation, which is thought to cause long-term activation of NFATc1. Since RANKL-induced Ca^2+^ oscillations are abolished in IP3 receptor (IP3R) knockout cells, Ca^2+^ release from the endoplasmic reticulum (ER) through IP3R channels is required to generate or sustain these Ca^2+^ oscillations [14]. Both the IP3R ligand IP3 and the protein kinase C activator diacylglycerol (DAG) are produced from the membrane phospholipid phosphatidylinositol 4,5-bisphosphate [PI(4,5)P2] by PLC. Therefore, metabolism of membrane phospholipids by PLC activation is critical for RANKL-induced Ca^2+^ signaling and subsequent NFATc1 activation during osteoclastogenesis.

**Fig. 1:**
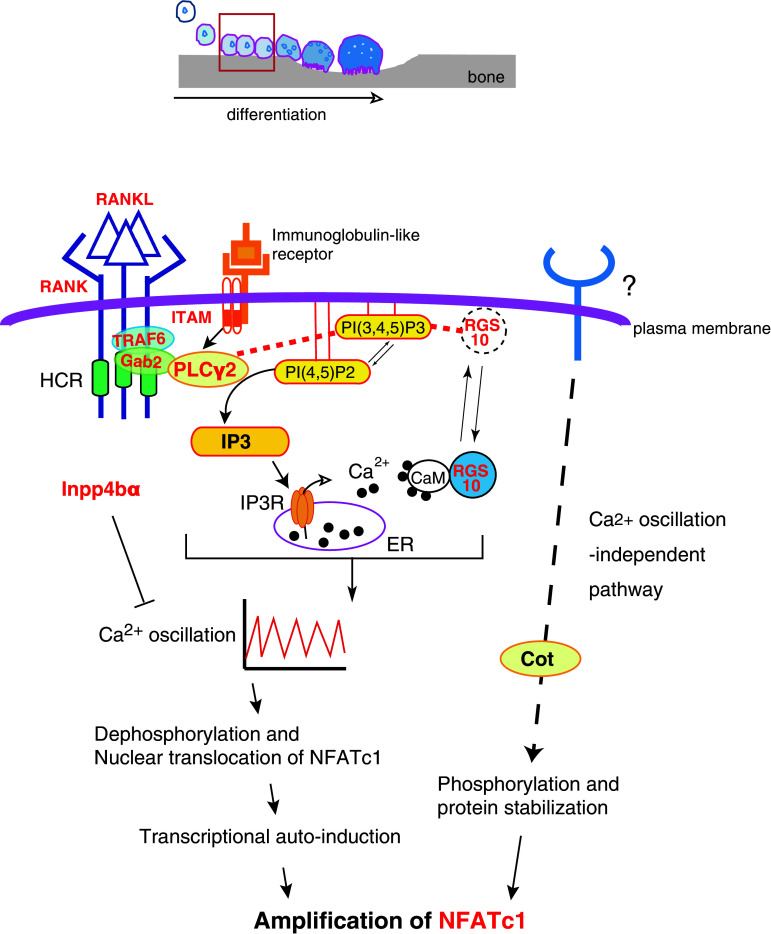
IP3 instigates the activation and amplification of NFATc1. Depicted above is a schematic illustrating the stages of osteoclast differentiation, including recruitment of progenitors to the bone surface, cell–cell fusion, formation of the actin ring and ruffled border and bone resorption. In the early phase of differentiation (*red-boxed*), osteoclastogenesis is triggered by RANKL–RANK signaling, which activates PLCγ2 to generate IP3 from PI(4,5)P2 in the plasma membrane. IP3 then stimulates calcium oscillations, which are required for subsequent activation of NFATc1. Knockout of the molecules in *red* have bone-related phenotypes largely because of impaired osteoclast differentiation (see the text for details). *Red dotted lines* indicate interactions between PIs and proteins. The *dotted arrow* indicates the Ca^2+^ oscillation-independent pathway to NFATc1 activation. This figure is modified from Kuroda et al., World Journal of Orthopedics (in press)

In concert with RANK signaling, immunoglobulin-like receptors such as osteoclast-associated receptor (OSCAR) and the triggering receptor expressed in myeloid cells 2 (TREM-2) transduce *Nfatc1* induction signals [15, 16]. Both receptors are associated with adaptor proteins such as DNAX-activation protein (DAP) 12 or the Fc receptor common γ subunit (FcRg) that possess the immunoreceptor tyrosine-based activation motif (ITAM) [17]. After ITAM tyrosine phosphorylation, a complex containing Bruton’s tyrosine kinase (Btk), tyrosine kinase expressed in hepatocellular carcinoma (Tec), the adaptor molecules B cell linker protein (BLNK) and Src homology 2 domain-containing leukocyte protein of 76 kD (SLP76) is formed that facilitates cooperation between RANK and ITAM signaling [18]. This combined signal leads to sustained PLCγ2 phosphorylation, suggesting that integration of RANK and ITAM signaling is required for the efficient activation of PLCγ2 and subsequent Ca^2+^ oscillations (Fig. 1). Furthermore, following elevation of intracellular Ca^2+^ but prior to calcium oscillations, *Nfatc1* transcription is enhanced by Ca^2+^/calmodulin-dependent kinase IV (CaMK IV). In turn, CaMK IV phosphorylates the cAMP response element-binding protein (CREB), inducing *Fos* expression [19].

The PLCγ family consists of the widely distributed PLCγ1 and the more restricted PLCγ2, which is primarily expressed by hematopoietic cells [20]. While *Plcg1* ^−/−^ mice cannot develop normally beyond embryonic day 8.5 [21], *Plcg2* ^−/−^ mice are viable but exhibit an osteopetrotic phenotype [22], indicating that PLCγ2 is required for osteoclastogenesis (Table 1). At a highly conserved region (HCR) in the RANK C-terminal tail, PLCγ2 forms a stimulus-dependent complex with the TRAF6 and Gab2 adapter proteins [23] (Fig. 1). An HCR deletion mutant of the CD40/RANK chimeric receptor does not alter NF-κB or MAPK activation but abolishes Ca^2+^ oscillations, indicating that HCR-mediated signaling is indispensable for sustained PLCγ2 activation and that sustained PLCγ2 activation is required to maintain Ca^2+^ oscillations [23].

**Table 1:**
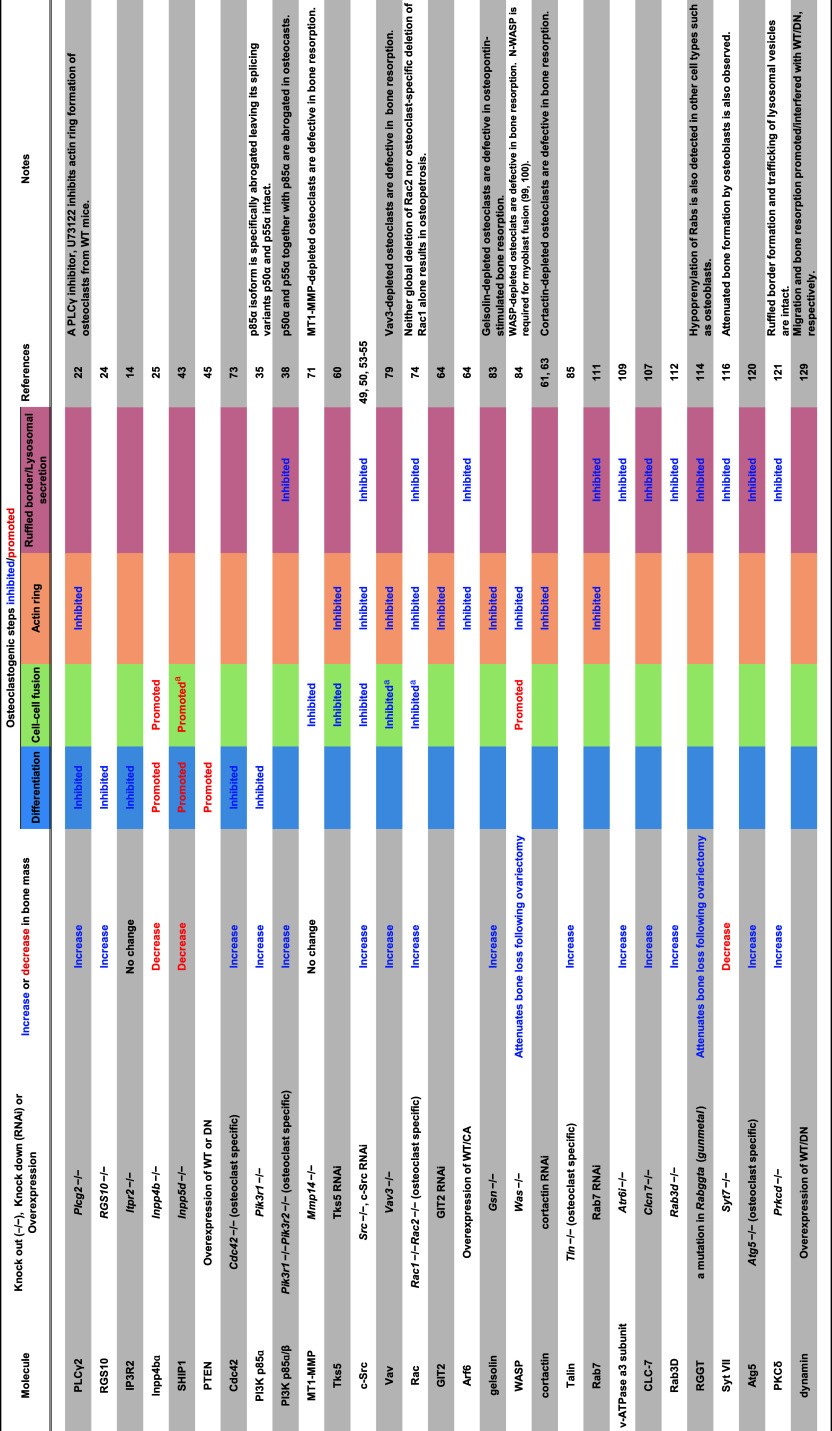
Bone and osteoclast-specific phenotypes that result from the manipulation of the expression of molecules participating in lipid-mediated osteoclast differentiation/function

The name of each molecule, the type of genetic manipulation, the bone mass, and osteoclast phenotypes resulting from knockdown or over-expression in cells or mice, and relevant references are shown

Blank boxes in the table represent “not determined”. *WT* wild type, *CA* constitutively active, *DN* dominant negative

^a^Apparent promotion or defect in cell–cell fusion cannot be distinguished from that in spreading

Calcium signaling during osteoclastogenesis is also controlled by PI-binding proteins. The regulator of G-protein signaling 10 (RGS10) competitively binds to phosphatidylinositol 3,4,5-trisphosphate [PI(3,4,5)P3] and this binding is required for RGS10 membrane localization and the subsequent activation of PLCγ2 and Ca^2+^ oscillations. The intracellular Ca^2+^ concentration shifts the balance between RGS10–PI(3,4,5)P3 and RGS10–Ca^2+^/CaM complexes and this may allow for self-sustaining Ca^2+^ oscillations through oscillatory regulation of PLCγ2 activation [24] (Fig. 1). Mice lacking RGS10 exhibit severe osteopetrosis due to defects in Ca^2+^ oscillations and reduced osteoclastogenesis in vivo, underscoring the importance of Ca^2+^ oscillations for NFATc1 activation and amplification during osteoclast differentiation [24] (Table 1). Inositol polyphosphate 4-phosphatase type IIα (Inpp4bα) can also modulate IP3-triggered Ca^2+^ signaling and subsequent osteoclastogenesis as suggested by the decreased bone mass observed in *Inpp4b* ^−/−^ mice [25] (Table 1). However, Inpp4bα efficiently hydrolyzes Ins(1,3,4)P3 but not IP3 in vitro [25]. Since Ins(1,3,4)P3 does not open purified IP3 receptors [26], the precise molecular mechanisms by which Inpp4bα ablation enhances Ca^2+^ signaling remains unknown. Nevertheless, as the human *INPP4B* was also identified as a susceptibility locus for osteoporosis [25], the balance among these different membrane inositol phospholipids could be a critical regulator of Ca^2+^ signaling and osteoclastogenesis.

In addition to Ca^2+^ oscillations, NFATc1 is also activated by an osteoblast-induced, Ca^2+^-independent pathway. When co-cultured with osteoblasts, cell–cell interactions increase NFATc1 protein levels even in osteoclast precursors derived from IP3R type2 and type3 (IP3R2/3) double knockout mice. Furthermore, osteoblasts promote osteoclast differentiation in the absence of detectable RANKL-induced Ca^2+^ oscillations [14] (Table 1). Phosphorylation-dependent protein stabilization of NFATc1 by Cot (Cancer Osaka thyroid) serine/threonine kinase, also known as tumor progression locus 2 (Tpl-2), partially explains Ca^2+^- and calcineurin-independent osteoclastogenesis [27]. Whether membrane phospholipids also contribute to Ca^2+^ oscillation-independent NFATc1 activation is unknown at present.

## PI3-kinases and their lipid products regulate osteoclast function

### PI3-kinase is activated downstream of osteoclastogenic stimuli

**Fig. 2:**
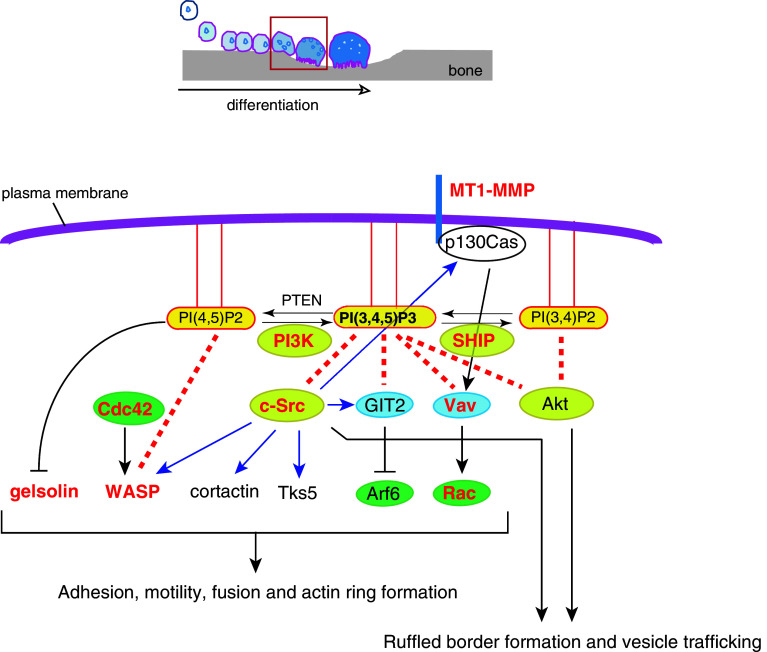
PI(3,4,5)P3 regulates osteoclast adhesion, motility, and ruffled border function. In the later phase of differentiation (*red-boxed* phase in the *top schematic*), signaling downstream of c-fms, RANK and α_v_β_3_ integrin activates PI3-kinase, triggering the production of PI(3,4,5)P3 in the plasma membrane. PI(3,4,5)P3 then recruits and/or activates cytosolic proteins, which are important for cytoskeletal rearrangement. Knockouts of the molecules in *red* have bone-related phenotypes because of functional defects in osteoclasts (see the text for details). *Blue arrows* indicate phosphorylation. *Red dotted lines* indicate interactions between PIs and proteins. Small GTPases are *encircled* in *green*, GEF/GAP in *blue*, and kinases/phosphatases in *yellow*

Once primed for differentiation into osteoclasts by IP3-triggered Ca^2+^ signaling and activation of NFATc1, a number of additional signaling molecules are activated that regulate osteoclast function. The PI3-kinase is one of the central downstream effectors of the M-CSF receptor c-fms [28, 29], RANK [30], and α_v_ *β*_3_ integrin [31] in osteoclasts. PI3-kinases can be classified into three groups: class I, which consists of regulatory and catalytic subunits such as p85 and p110; class II kinases that do not require adaptor subunits; and class III kinases with a catalytic subunit p110 that shares homology with the yeast PI3-kinase Vps34p. Activation of class I PI3-kinase downstream of RANK leads to the production of PI(3,4,5)P3 from PI(4,5)P2. Class I and II PI3-kinases produce PI(3,4)P2 from PI(4)P; alternatively, PI(3,4)P2 can be generated by dephosphorylation of PI(3,4,5)P3 by PI(3,4,5)P3 5-phosphatases such as Src homology 2-containing inositol-5-phosphatase 1 (SHIP1) [32] (Fig. 2). In general, PI(3,4,5)P3 and PI(3,4)P2 serve as stimulants of cell proliferation, survival, and directional migration by anchoring effectors like Akt [32]. PI3-kinase activity can be quenched by generation of PI(4,5)P2 from PI(3,4,5)P3 through hydrolysis by a tumor suppressor gene product, phosphatase and tensin homolog deleted from chromosome 10 (PTEN) [33] (Fig. 2). Thus, PI3-kinase is bidirectionally regulated by a number of proteins.

The PI3-kinase is required for activation of Akt and MAPK [34], so it is not surprising that mice lacking the p85α subunit of class I PI3-kinase exhibit impaired osteoclast proliferation and maturation [35] (Table 1) as well as of impaired B cell development and activation [36, 37]. A study using osteoclast-specific p85α/β double knockout mice demonstrates that PI3-kinase-dependent activation of Akt is essential for ruffled border formation and vesicle transport [38] (Table 1). Knockouts of the p110α or p110β catalytic subunit (*Pik3ca* ^−/−^ or *Pik3cb* ^−/−^, respectively) result in embryonic lethality [39, 40], while p110γ knockouts (*Pik3cg* ^−/−^) are viable but exhibit defects in T cell proliferation and function as well as reduced neutrophil migration and chemotaxis [41]. Although a study using a specific inhibitor suggests a dominant role for p110α in osteoclast differentiation [42], detailed skeletal analysis of conditional knock out of these class I catalytic subunits and deletion of other classes of PI3-kinase genes will be required to clarify the role of each PI3-kinase class and isoform in osteoclastogenesis. In contrast to mice lacking the p85α PI3-kinase subunit, *Inpp5d* ^−/−^ mice lacking SHIP1 exhibit the reverse phenotype, with more numerous and larger osteoclasts that are hypersensitive to M-CSF and RANKL, as well as less apoptotic and hyper-resorptive, resulting in osteoporosis [43] (Table 1). Another study found that the granulocyte–macrophage progenitors of *Inpp5d* ^−/−^ mice show enhanced proliferative potential [44]. Similarly, PTEN negatively regulates osteoclast differentiation [45] (Table 1). These results strongly suggest that PI(3,4,5)P3 is a primary inducer of osteoclastogenesis (Figs. 1 and 2). It would be of great interest to test if direct addition of liposomes containing PI(3,4,5)P3 to osteoclast precursors accelerates osteoclastogenesis. However, it is possible that the spatiotemporal production of PI(3,4,5)P3 must be tightly controlled to properly drive osteoclastogenesis, given the role of PI(3,4,5)P3 as an anchor and the functional segregation of different membrane compartments of the multinuclear osteoclast.

### PI- and raft-dependent c-Src activity is required for osteoclastogenesis

Locally produced PI(3,4,5)P3 and PI(3,4)P2 recruit cytosolic proteins to the plasma membrane. For example, direct interaction between PI(3,4,5)P3 and the Src-homology 2 (SH2) domains of PI3-kinase or the ubiquitous tyrosine kinase c-Src stimulates the formation of a protein complex containing PI3-kinase or c-Src and gelsolin [46]. Membrane targeting of c-Src is also aided by covalent binding of the 14-carbon fatty acid myristate and by the basic amino acid residues at the c-Src N-terminal [47]. In addition, specialized membrane microdomains enriched with cholesterol and sphingolipids (called lipid rafts) are platforms for enrichment of c-Src activity [48]. Membrane-associated c-Src is indispensable for osteoclast functions, as evidenced by the observation that osteoclasts from *Src* ^−/−^ mice manifest impaired formation of actin rings and reduced bone resorption activity, leading to severe osteopetrosis [49, 50] (Table 1). Moreover, this phenotype was not mimicked by deletion of other Src-family kinases [51], indicating the importance of c-Src and its specific binding proteins and substrates in osteoclastogenesis although Hck partially compensates for c-Src as revealed by *Hck* ^−*/*−^ *Src* ^−/−^mice [52]. Indeed, c-Src has been shown to regulate cytoskeletal dynamics [53] as well as cell spreading, cell–cell fusion and ruffled border formation [54, 55], seminal early events in osteoclastogenesis vital for subsequent bone resorption. Among these c-Src binding partners are FAK [56, 57], p130Cas [58], WASP [59], Tks5 [60], cortactin [61–63] and GIT2 [64] proteins that mediate adhesion, podosome/fusion-competent protrusion and actin ring formation in osteoclasts (Fig. 2; Table 1). Furthermore, c-Src is also localized at the intercellular vesicular membranes and ruffled border, where it contributes to the secretion of bone-degrading acids and enzymes [65–68].

### Cytoskeletal reorganization by small GTPases is controlled by PI(4,5)P2 and PI(3,4,5)P3

The coalescence and fusion of osteoclasts requires the activation of Rho-family GTPases and molecules that rearrange the actin cytoskeleton [69, 70]. A membrane-type 1 matrix metalloproteinase (MT1-MMP)—p130Cas—Rac signaling pathway was recently shown to be indispensable for this process [71](Fig. 2). Bone marrow cells from *Mmp14* ^−/−^ mice lacking MT1-MMP are still committed to the osteoclast lineage as they express osteoclast genes like *Nfatc1* and the TRAP gene *Acp5* but exhibit defects in migration and cell–cell fusion [71] (Table 1). Small GTPases of the Rho and Arf family, such as Rho, Rac, Cdc42 and Arf6, are also recruited and activated/inactivated by PIs on the membrane to regulate osteoclast differentiation and bone resorption [64, 72–74] (Table 1). Indeed, these proteins are central regulators of cytoskeletal remodeling, protrusion formation, and membrane trafficking [75, 76]. Activity is stimulated by guanine nucleotide exchange factors (GEFs) such as the Rac activating Vav family proteins and decreased by GTPase-activating proteins (GAPs), such as Arf6 inhibitors GIT2 or centaurin. These GEFs and GAPs often possess pleckstrin homology (PH) domains through which they directly interact with PI(4,5)P2, PI(3,4)P2 and/or PI(3,4,5)P3 [77, 78]. The GEF Vav3 is crucial for Rac activation and subsequent cytoskeletal rearrangement in osteoclasts as evidenced by osteopetrosis in *Vav3* ^−/−^ or *Vav1* ^−/−^ *Vav3* ^−/−^ mice [79] (Table 1). Osteoclasts from these mice do express osteoclast gene products in response to M-CSF and RANKL, but circumferential podosome formation, cell–cell fusion and bone resorption are impaired [79]. Osteoclasts with reduced expression of the c-Src substrate and Arf6 inhibitor GIT2 induced by RNA interference (RNAi) appear to differentiate normally but lack actin rings [64] (Table 1). While GIT2 lacks the PH domain allowing direct interaction with PIs, it is activated by PI(3,4,5)P3 [80], and this interaction suppresses Arf6 activity. The GTPase Arf6 is required for the formation of membrane protrusions such as invadopodia in cancer cells by promoting endosomal recycling and Rac-mediated cytoskeletal remodeling [76, 81]. Therefore, excessive Arf6 activity in GIT2 knockdown osteoclasts may allow for the formation of circumferential podosomes, which are structures analogous to invadopodia, but obstruct later actin ring formation. Analogous to GIT2 function in osteoclast differentiation, previous studies reported that GIT2 regulates the directional chemotaxis of neutrophils and that the loss of GIT2 in vivo leads to immunodeficiency [82]. Centaurin-α2, another GAP for Arf6 with a PH domain, is also essential for Arf6-dependent cytoskeletal remodeling [78], thereby supporting the importance of the PI(3,4,5)P3-Arf6 pathway in osteoclast maturation (Fig. 2).

The cytoskeletal proteins gelsolin, villin, cofilin, and profilin, which sever or depolymerize actin filaments in vitro, are inactivated by PI(4,5)P2, a PI synthesized by phosphatidylinositol 4-phosphate 5-kinase (PI4P-5 kinase). Conversely, several cytoskeletal proteins that bundle actin filaments or link them to the plasma membrane, including vinculin, talin, ezrin/radixin/moesin (ERM) proteins, WASP/N-WASP and α-actinin, are activated by PI(4,5)P2 and/or PI(3,4,5)P3. Consequently, membrane PIs also control cytoskeletal dynamics and osteoclast functions by regulating the activities of these cytoskeletal proteins. Whole animal knockout of gelsolin (*Gsn* ^−/−^) [83] or Wasp (Was^−/−^) [84], or osteoclast-specific knockout of Talin (*Tln* ^−/−^) [85] increases mouse bone mass or attenuates bone loss following ovariectomy (Table 1). *WAS* deletion also eliminates the formation of podosome clusters in human primary macrophages [86], thus explaining the impaired migration and invasion of macrophages in patients with Wiskott–Aldrich syndrome (WAS). On the other hand, *Was* ^−/−^ osteoclasts exhibit a greater number of nuclei per cell [84], indicating that cell–cell fusion is enhanced. Both podosomes and fusion-competent protrusions (see below) require cytoskeletal remodeling and membrane deformation. However, according to the knockout phenotype, WASP is exclusively required for podosomes but not for fusion-competent protrusions. In the absence of WASP, additional quantities of actin-regulatory and membrane-deforming molecules interacting with WASP, e.g., Arp2/3 complex and Cdc42, are thought to be used for fusion-competent protrusions, instead of being used for podosomes. This may explain the enhanced cell–cell fusion in *Was* ^−*/*−^ osteoclasts [84]. It is plausible that the formation of perpendicular actin-rich membrane protrusions like podosomes or horizontal fusion-competent protrusions depends on a balance between PI-regulated complementary cytoskeletal GTPases that either promote actin polymerization or bundling and membrane association. Tropomyosin (Tm) stabilizes actin filaments by functionally antagonizing depolymerization or severing factors such as gelsolin and cofilin [87]. Expression of Tm-2 and Tm-3 is induced in the late phase of osteoclastogenesis and reduced expression or overexpression results in altered spreading, motility, and resorption of osteoclasts [88]. Therefore, a balance of activity among cytoskeletal proteins described above may explain osteoclasts’ resorptive/migratory cycle (polarization/depolarization cycle).

### Cell–cell fusion is achieved by fusion-competent protrusions downstream of PI3-kinase

Osteoclasts must overcome a significant energy barrier for the fusion of apposing lipid bilayers given that plasma membranes do not spontaneously fuse. In vitro protein-free experiments indicate that lipid bilayer fusion involves the following steps: establishment of close contact between the bilayers so that they become at least partially dehydrated, formation of highly curved protrusions between bilayers to expose an unstable outer leaflet, resulting in hemifusion, and final formation of a fusion pore, a process that requires the lateral tension concomitant with local or global membrane expansion [89–92]. Osteoclast-specific fusogens such as DC-STAMP [93], OC-STAMP [94], macrophage fusion receptor (MFR) [95], v-ATPase V0 subunit d2 [96], and CD9 in lipid rafts [97] are thought to lower the first intermediate energy barriers by tightly tethering the opposing plasma membranes. Proteins that have membrane-deforming activity or those inducing membrane expansion could then contribute to hemifusion and fusion pore formation. In fact, circumferential podosomes, but not mature actin rings, may supply the lateral tension necessary to drive fusion pore opening [91]. When probed with markers of PI-binding domains, the fusion sites are often enriched with products of PI3-kinases [60]. Inhibition of PI3-kinase activity only during the period of highest fusion frequency results in fusion defects, while the expression of osteoclast genes are unaffected [60]. The phox homology (PX) domain adaptor protein Tks5, known to regulate invadopodia formation in cancer cells [98], was found to act downstream of PI3-kinase and Src in promoting cell–cell fusion. Reduced expression of Tks5 results in the loss of circumferential podosomes and cell–cell fusion [60] (Table 1), which is in accordance with recent findings showing that N-WASP-dependent actin-rich protrusive structures are also key drivers of myoblast fusion [99, 100]. Alternatively, circumferential podosome expansions in osteoclasts often accompany tiny membrane protrusions [101] that might be generated by membrane-deforming proteins such as Bin-Amphiphysin-Rvs161/167 (BAR) domain superfamily proteins [102]. For this reason, we refer to the protrusions observed during osteoclast fusion as *fusion*-*competent protrusions*. Studying the shared and distinct fusion mechanisms in multiple biological processes may soon provide a more complete and clear picture of osteoclast fusion.

## Efficient bone resorption is achieved through ruffled border formation and intracellular membrane trafficking

**Fig. 3:**
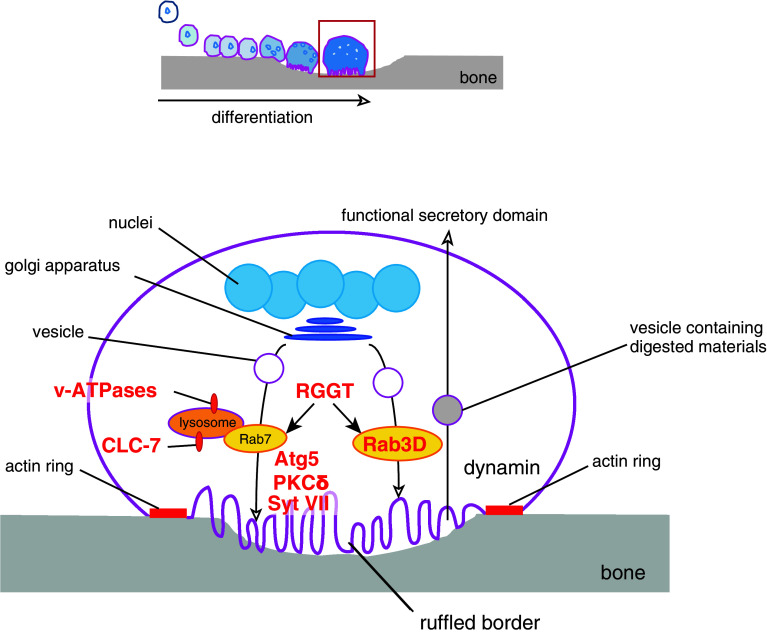
Vesicular trafficking enables bone resorption. In the resorbing phase (*red-boxed* phase in the *top schematic*), Rab GTPases mediate vesicle trafficking, while the fusion of vesicles with the ruffled border to release acids and hydrolases is mediated by Syt VII and Atg5. Dynamin-mediated endocytosis clears degraded materials. Knockouts or mutations of the molecules in *red* have bone-related phenotypes because of functional defects in osteoclasts (see text for details)

Fused osteoclasts reorganize their actin cytoskeletons and eventually form F-actin-rich adhesive structures called actin rings on the ventral membranes contacting the bone surface [6]. The membrane area enclosed by the actin ring, termed the ruffled border, secretes protons and hydrolases that solubilize and digest inorganic and organic bone matrix. The ruffled border was originally found to be the site of lysosomal secretion [103, 104] and defects in its formation were reported in osteoclasts from patients with malignant juvenile osteopetrosis [105]. The a3 subunit of v-ATPases and the Cl^−^/H^+^ antiporter CLC-7 localize at both the lysosomes and ruffled border, where they function to acidify secreting lysosomes and the space between the ruffled border and bone surface [106, 107] (Fig. 3). Notably, mutations in the gene encoding the a3 subunit of v-ATPases (*OC116*) or in the gene encoding CLC-7 (*CLCN7*) are reported in malignant juvenile osteopetrosis [107, 108]. Similarly, disruption of *Atr6i*, the gene encoding the a3 subunit of v-ATPases in mice, or mouse *Clcn7* causes osteopetrosis [107, 109] (Table 1). Furthermore, the small GTPase Rab7, which regulates vesicle fusion to late endosomes or lysosomes, also localizes to the ruffled border [110] (Fig. 3). Reduced expression of Rab7 impairs actin ring formation, ruffled border formation and bone resorption in vitro [111], while multinucleation (cell–cell fusion) is unaffected (Table 1). Rab3D is another Rab GTPase that is essential for osteoclast function as revealed by *Rab3d* ^−/−^ mice that exhibit an osteopetrotic phenotype [112] (Table 1). Osteoclasts from these mice form disturbed ruffled borders with normal actin rings [112] (Table 1). However, the vesicular trafficking pathway mediated by Rab3D appears different from that mediated by Rab7 as judged by their distinct subcellular localization (Fig. 3). Generally, Rab GTPases function only when the C-terminal cysteine(s) are covalently linked to a farnesyl or geranylgeranyl moiety (called prenylation). The importance of prenylation for Rab GTPase function and osteoclast-mediated bone resorption is demonstrated by *gunmetal* mice, which have an autosomal recessive mutation in the gene encoding Rab geranylgeranyl transferase (RGGT), resulting in a 70 % reduction in GTPase activity [113] and osteoclasts with normal cytoskeletal architecture but reduced resorptive activity [114] (Table 1).

Synaptotagmin VII (Syt VII), a member of the synaptotagmin family that mediates Ca^2+^-triggered fusion of cytoplasmic/synaptic vesicles to the plasma membrane [115], localizes at the ruffled border, and promotes the secretion of lysosomal contents [116] (Fig. 3). Osteoclasts deficient in Syt VII (*Syt7* ^−/−^) fail to localize cathepsin K to the resorptive microenvironment or form ruffled borders; however, the bone density of *Syt7* ^−/−^ mice is actually reduced partly because of attenuated bone formation by osteoblasts [116] (Table 1). Unlike many other phases of osteoclastogenesis, proteins regulating the formation of the ruffled border are largely unknown. In this regard, autophagic proteins that regulate lysozyme secretion in intestinal Paneth cells [117], insulin secretion in pancreatic *β* cells [118], or degranulation of mast cells [119] are strong candidate effectors of ruffled border formation and extracellular secretion by osteoclasts. Indeed, osteoclast-specific deletion of autophagy-related (Atg) protein Atg5 leads to increased bone mass and alleviates bone loss caused by ovariectomy [120] (Fig. 3; Table 1). Further, the conjugation of the mammalian Atg8 homolog LC3 with phosphatidylethanolamine (PE) is indispensable for the proper trafficking of cathepsin K to the ruffled border [120]. Both Syt VII and Atg5 are required for ruffled border formation/maturation and vesicle-membrane fusion but not for actin ring formation [116, 120] (Table 1). Protein kinase Cδ is another likely participant in cathepsin K secretion. Mice deficient in *Prkcd* ^−/−^ are osteopetrotic and protected from bone loss induced by ovariectomy [121] (Table 1). Intriguingly, impaired cathepsin K secretion in *Prkcd* ^−/−^ osteoclasts is independent of ruffled border formation and trafficking of lysosomal vesicles [121] (Table 1). Therefore, the DAG-PKCδ pathway may promote cathepsin K secretion through alternate mechanisms.

While digesting the bone matrix, osteoclasts must properly dispose of large amounts of calcium, phosphate, and digested collagen that would otherwise rise to cytotoxic levels. Osteoclasts transport these products and transcytose the vesicles containing these materials to the apical region of the plasma membrane, called functional secretory domain, into the extracellular space [122, 123] (Fig. 3). The ruffled border is thus the site of extensive endocytic activity and expresses known endocytic proteins like clathrin, AP-2 and dynamin [124, 125]. Exogenous small tracer molecules rapidly enter the osteoclast and are found at the ruffled border within minutes [126]. Therefore, at least some endocytosis from the ruffled border is thought to be receptor-independent and non-specific, resembling macropinocytosis. Dynamin, a PH domain-containing GTPase essential for podosome formation and endocytosis, is pivotal for the coat-dependent specific uptake [127, 128] (Fig. 3). Overexpression of dynamin stimulates osteoclast migration and resorption and this stimulation depends on the presence of dynamin GTPase activity [129] (Table 1). Dynasore, a specific inhibitor of dynamin [130], may be a useful agent for treating osteoporosis if selectively delivered to osteoclasts.

It has been suggested that raft-dependent membrane trafficking from the ventral or apical membrane is necessary to maintain a functional ruffled border [110, 131], but it is still unclear as to how osteoclasts organize and segregate the functionally distinct membrane regions or as to how regions like the ruffled border and apical membrane coordinate membrane recycling so that exocytosis and endocytosis are optimized.

## Conclusions

In this review, we presented evidence demonstrating that membrane lipids, particularly PIs, are crucial for osteoclast differentiation and bone resorption. As osteoclastogenesis is largely dependent on IP3-mediated Ca^2+^ oscillations, signaling pathways that lead to IP3 production are of special importance. Indeed, knockout of various molecules in Ca^2+^- or IP3-dependent pathways result in osteopetrotic phenotypes because of impaired osteoclast differentiation and bone resorption (Fig. 1; Table 1). Once committed, remodeling of both the actin cytoskeleton and the plasma membrane drives the morphological changes associated with osteoclastogenesis. During morphological transformation and maturation, there are three major barriers to overcome according to knockout or knockdown phenotypes: (1) cell–cell fusion, (2) actin ring formation, and (3) ruffled border formation. (1) To overcome the energy barrier of membrane fusion, fusogens on the plasma membrane and efficient migration and formation of fusion-competent protrusions are required. Production of PI(3,4,5)P3 and/or PI(3,4)P2 on the membrane triggers migration and protrusion formation in pre-fusion osteoclasts (Fig. 2; Table 1). Without cell–cell fusion, mononuclear osteoclasts can still proceed to the next barrier. (2) Actin ring formation requires actin regulatory molecules that act to stabilize dense actin bundles (Fig. 2 and Table 1). Without actin rings, osteoclasts cannot proceed to the next barrier. The actin ring acts to segregate hydrolytic enzymes, acids, and toxic digestive products from the extracellular environment. Actin ring formation is also dependent on inositol phospholipids that regulate a variety of small GTPases associated with the membrane and cytoskeleton. (3) The ruffled border of osteoclasts allows these cells to efficiently release degradative enzymes and resorb digestion products for recycling by membrane trafficking and vesicle–membrane fusion (Fig. 3; Table 1).
